# Mucosal Tuft Cell Density Is Increased in Diarrhea-Predominant Irritable Bowel Syndrome Colonic Biopsies

**DOI:** 10.3389/fpsyt.2020.00436

**Published:** 2020-05-15

**Authors:** Jessica Aigbologa, Maeve Connolly, Julliette M. Buckley, Dervla O'Malley

**Affiliations:** ^1^APC Microbiome Ireland, Cork, Ireland; ^2^Department of Physiology, University College Cork, Cork, Ireland; ^3^Department of Surgery, University College Cork, Cork, Ireland; ^4^Mater Private Hospital, Cork, Ireland

**Keywords:** interleukin-25, irritable bowel syndrome, helminths, Wistar Kyoto, brush cells, Doublecortin Linked Kinase-1, DCAMKL1, chronic stress

## Abstract

Tuft cells are rare chemosensory sentinels found in the gut epithelium. When triggered by helminth infection, tuft cells secrete interleukin-25 (IL-25) basolaterally and subsequently evoke an immune response. Irritable bowel syndrome (IBS) is a common and heterogeneous disorder characterized by bowel dysfunction and visceral pain sensitivity. Dysfunctional gut-brain communication and immune activation contribute to the pathophysiology of this disorder. The study aims were to investigate changes in tuft cell density in non-post-infectious IBS patients. Immunofluorescent labeling of DCLK1-positive tuft cells was carried out in mucosal biopsies from the distal colons of diarrhea and constipation-predominant IBS patients and healthy controls. Tuft cell numbers were also assessed in animal models. Concentrations of interleukin-25 (IL-25) secreted from colonic biopsies and in plasma samples were analyzed using an immunoassay. The density of tuft cells was increased in diarrhea—but not constipation-predominant IBS patient colonic biopsies. Biopsy secretions and plasma concentrations of IL-25 were elevated in diarrhea—but not constipation-predominant IBS participants. Tuft cell hyperplasia was detected in a rat model of IBS but not in mice exposed to chronic stress. Tuft cell hyperplasia is an innate immune response to helminth exposure. However, the patients with diarrhea-predominant IBS have not reported any incidents of enteric infection. Moreover, rats exhibiting IBS-like symptoms displayed increased tuft cell density but were not exposed to helminths. Our findings suggest that factors other than helminth exposure or chronic stress lead to tuft cell hyperplasia in IBS colonic biopsies.

## Introduction

Tuft cells are rare differentiated epithelial cells, anatomically and functionally distinct from other border cells in the gastrointestinal (GI) tract (1). Characterized by long, blunt microvilli, pear-shaped tuft cells are scattered along the crypt-villus axis (2). Uniquely, they express a microtubule linked protein known as Doublecortin Linked Kinase-1 (3) (DCLK1, also known as DCAMK1 (4)] and contain axial bundles of actin filaments supporting the microvilli (5, 6). The chemosensory activity and intimate physical contact between tuft cells and enteric nerves (7, 8) suggests a role in regulating gut motility and absorpto-secretory function. Tuft cells could also act as cross-epithelial signal transducers (8), informing the host nervous system of changes in the luminal environment. Parasitic infections, in particular (9), uniquely stimulate release of immune cytokines, such as interleukin (IL)-25 (also known as IL-17E), from tuft cells (10–12). IL-25, in turn, induces secretion of IL-13 from stromal group 2 innate lymphoid cells (ILC2), which promote release of IgE, eosinophilia, goblet cell hyperplasia (13) and, in a feed forward circuit, tuft cell hyperplasia (14).

Irritable bowel syndrome (IBS), a prevalent, chronic and heterogeneous functional bowel disorder, is characterized by abdominal pain, bloating and altered bowel motility (15). Prevalence of IBS is ~7–18% of the worldwide population (16), and this includes a subset referred to as post-infectious IBS (PI-IBS) patients, who develop intestinal dysfunction following infectious enteritis (17). Indeed, prior GI infection is a strong predictor of developing IBS (18), with one in ten patients believing their IBS symptoms emerged subsequent to an infectious illness (17). Infection with protozoans as opposed to bacteria conferred a greater risk of developing IBS following resolution of the infection (19).

Although it is plausible that tuft cell numbers could be elevated in patients with PI-IBS, the majority of IBS patients do not report prior GI infection. Rather, a significant proportion of these patients, who may be sub-categorized with diarrhea (IBS-D), constipation (IBS-C) or alternating subtypes of IBS, experience co-morbid anxiety and depressive disorders (20). Thus, it is generally accepted that dysfunction of the bi-directional gut-brain axis underlies symptoms in these patient groups. This study aims to quantify expression of tuft cells in colonic samples from IBS patients who have not, to their knowledge, had prior intestinal enteritis. Tuft cell density was assessed in non-PI IBS patients, in a stress-sensitive rat model of IBS and in mice exposed to a chronic stressor to determine if stimuli other than exposure to parasites contributes to IBS pathophysiology.

## Materials and Methods

### Ethical Approval

The protocol for collecting biopsies and blood samples from IBS patients and healthy control volunteers was approved by the University College Cork Clinical Research Ethics Committee (ECM 4 (r) 010316) and was carried out in the Mater Private Hospital, Cork. Informed consent was obtained from all participants.

Experiments using animal tissue were all in full accordance with the principles of the European Community Council Directive (86/609/EEC) as well as the local University College Cork animal ethical committee (#2011/015).

### Human Colon Biopsy and Plasma Collection

Patients attending the General Surgery Clinic at the Mater Private Hospital, Cork, Ireland were recruited for the study. Males and females aged between 18 and 65 years of age and able to provide written informed consent were enrolled. Inclusion criteria for IBS patients included confirmed clinical diagnosis of IBS that satisfied Rome III criteria for IBS. No PI-IBS patients were included in this study. Biopsies from age and weight-matched healthy controls were taken from patients undergoing routine colonoscopies that were in good health and negative for bowel disease. Exclusion criteria for participation included acute or chronic co-existing illness, recent unexplained bleeding or prior GI surgery (apart from hernia repair and appendectomy), coeliac or other GI disease, psychiatric disease, immunodeficiency, bleeding disorder, coagulopathy, a malignant disease or any concomitant end-stage organ disease. Subjects were also excluded if they were taking any experimental drugs or if the subject had taken part in an experimental trial less than 30 days prior to this study. Mucosal biopsies from the distal colon were taken from fasting patients at the same time as obtaining a matched serum sample. Samples were assigned a study number, with the key held only by the treating surgeon, so as to preserve patient confidentiality in accordance with the study protocol. The secretory products from biopsies incubated in Dubellco's Modified Eagle Medium (Sigma Aldrich, UK, overnight, 37°C) were used to measure local tissue concentration of interleukin-25 (IL-25)/hu-17E. Mucosal biopsies were subsequently fixed overnight in 4% paraformaldehyde at 4°C, cryoprotected in 30% sucrose and stored at −80°C for immunofluorescent staining.

### Animals and Tissue Collecting

Male Sprague Dawley (SD) and Wistar Kyoto (WKY) rats, > 8 weeks of age, were purchased from Envigo, Derbyshire, UK. Given that hormonal cycles in the female are associated with exacerbation of IBS-like symptoms, we used male rodents in this study such that the additional complexity of changing female hormone levels was not a factor in the studies. The Animals were group-housed four per cage and maintained on a 12/12-hour dark-light cycle with a room temperature of 22 ± 1°C with food and water *ad libitum*. Rats were euthanized by CO_2_ overdose and perforation of the diaphragm.

Male adult mice (C57Bl/6J, The Jackson Laboratory, Maine, US) were bred in-house (Biological Service Unit, University College Cork, Ireland). Prior to social defeat sessions (~1 week) mice were singly housed. Singly-housed adult male CD1 mice (Envigo, UK) were used as aggressors for the chronic social defeat stress procedure. Mice were maintained on a 12/12-hour dark-light cycle with a room temperature of 22 ± 1°C with food and water *ad libitum*. Mice were sacrificed by cervical decapitation.

Chronic social defeat stress in these mice has been previously described (21). In brief, mice assigned to the chronic social defeat stress group underwent 10 consecutive days of stress. The same researcher carried out all interventions. All defeat sessions were carried out in the mornings during the light cycle. CD1 aggressor mice were selected based on the shortest latency to attack another CD1 mouse. Test mice were subjected to a different CD1 aggressor mouse each day over the study period. Exposure of the test mouse to the aggressive CD1 mouse lasted until the first attack, expression of submissive posturing or until 5min had passed, whichever happened first. The test and CD1 aggressor mice were then separated by a perforated transparent barrier for 2h. The separator was subsequently removed and, after another defeat, mice were transferred back to their home-cage. Control mice were handled but remained in their home-cages over the course of the stress.

The distal colon (< 4 cm from anus) from both rats and mice were isolated and placed in ice-cold 95% O_2_/5% CO_2_ bubbled Krebs saline solution consisting of (in mmol/L) NaCl, 117; KCl, 4.8; CaCl_2_, 2.5; MgCl_2_, 1.2; NaHCO_3_, 25; NaH_2_PO_4_, 1.2; and D-glucose, 11. Colonic samples were fixed in 4% paraformaldehyde at 4°C overnight. The samples were then cryoprotected in 30% sucrose and snap frozen at −80°C.

### Mesoscale Discovery Biomarker Assay

An immunoassay (U-PLEX Human IL-17E/IL-25 Assay, MesoScale Discovery, Gaithersburg, MD, USA) was carried out to determine the concentration of IL-17E/IL-25 in plasma and supernatant samples of IBS patients and healthy control samples (dynamic range: 0.58–9,200 pg/ml). The assay was run in triplicate and an electrochemiluminescent detection method was used to measure protein levels in the samples. The plates were read using MesoScale Discovery plate-reader (MESO QuickPlex SQ 120). A calibration curve was generated using standards, and cytokine concentrations were determined from the curve.

### Immunofluorescence and Confocal Microscopy

Cross-sections of rat and mouse distal colon and human distal colonic biopsies, fixed in 4% paraformaldehyde (4°C, overnight), were cryo-sectioned (10 µm in thickness, Leica Biosystems, Wetzler, Germany) and mounted on glass slides (VWR, Dublin 15, Ireland). Rodent cross-sections or human mucosal biopsies were permeabilized with 0.1% Triton X-100 and blocked with 1% donkey serum (Sigma Aldrich, UK). Colonic tissue was immunolabeled with anti-DCLK1 (1:100, overnight at 4°C, anti-DCAMKL1 polyclonal rabbit antibody, Abcam, Cambridge, UK) and a complimentary TRITC-conjugated fluorophore (1:250, 2 h at room temperature, Jackson ImmunoResearch Europe Ltd., Cambridgeshire, UK). This primary antibody recognizes a protein of the predicted size and is blocked by using a DCAMKL1 peptide (22). No non-specific fluorescence was detected in control experiments where tissues were incubated with anti-DCLK1 in the absence of secondary antibodies or secondary antibodies alone. As tuft cells have a unique arrangement of cytoskeletal components, colonic samples were co-stained with a cytoskeletal marker, Phalloidin-iFluor 488-Cytopainter (1:1,000, Abcam, Cambridge, UK), which was prepared in 1% bovine serum albumin in phosphate buffered saline solution (PBS, (in mM): NaCl 137, KCl 2.7 and Na_2_HPO_4_ 10 at pH 7.4). Tissue sections were mounted using Dako-fluorescent mounting medium containing DAPI (Agilent Pathology Solutions Santa Clara, California, USA) and a coverslip placed over all tissue. Images were captured using a FVl0i-Olympus-confocal microscope with Fluoview software (FV10i-SW, Olympus Europe, Hamburg, Germany). At least three different biopsy slices from six different participants per group were compared in the human study. In the animal studies, at least three different cross-section slices from three different animals per group were compared. Analysis was carried out independently by two different researchers and the mean number of cells from each was calculated.

### Statistical Analyses

Data was analyzed using GraphPad prism for windows (version 7). Data were plotted as box and whisker plots with 95% confidence intervals. Data were compared using paired two-tailed Student's tests or One-way or repeated measures ANOVA with Tukey post-hoc test, as appropriate. P values of <0.05 were considered significant.

## Results

### Tuft Cell Density Is Elevated in IBS-D Patient Biopsies

Samples from healthy controls (HC, n = 6 (three males, three females)) were compared with samples from diarrhea-predominant (IBS-D, n = 6 (one male, five females)) and constipation-predominant (IBS-C, n = 6, (two males, four females)) participants. HC and patient participants were similar in terms of ethnicity (all Caucasian), age—(44.7 ± 4.56 (HCs) versus 40 ± 3.91 (IBS) years, p >0.05) and weight—(72.9 ± 13.95 (HCs) versus 71.83 ± 10.97 (IBS) kg, p >0.05). Gastrointestinal symptoms, such as bloating, abdominal pain and altered bowel habit were consistent with their categorization into the appropriate IBS subtype, as determined by Rome III criteria for diagnosing IBS. Mood disorders were reported in one IBS-D (depression), two IBS-C (depression and/or anxiety) but no HC participants.

**Figure 1:**
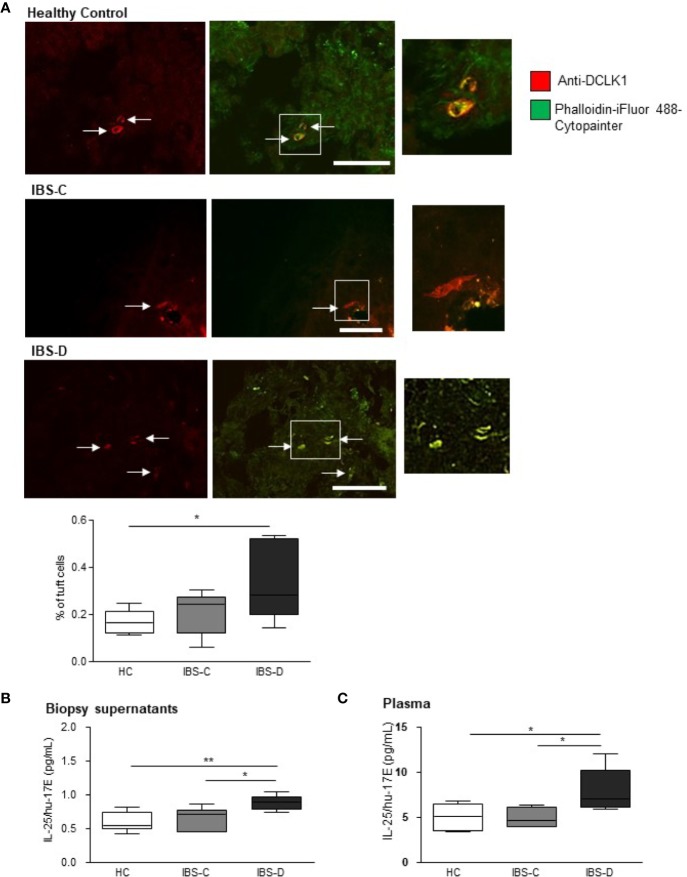
Tuft cell density and IL-25 secretion is elevated in IBS-D colonic mucosa. **(A)** The representative immunofluorescent images and box and whisker plots of pooled data illustrate the density of DCLK1-labeled tuft cells as a percentage of the total DAPI-stained epithelial cells in mucosal biopsies from healthy patients and patients with constipation- (IBS-C) or diarrhea-predominant (IBS-D) IBS. Scalebar: 50 µm. **(B)** The pooled data shows that colonic biopsies and **(C)** plasma samples from human IBS-D patients secrete more IL-25 than other groups. * and ** indicate p <0.05 and p <0.01, respectively.

Triple-labeling of human colonic biopsies with an antibody against the gastrointestinal tuft cell marker, doublecortin-like kinase 1 protein (DCLK1, red staining) (23), a cytoskeletal marker (green staining) and the nuclear stain, DAPI, facilitated counting of tuft cells as a percentage of total DAPI-labeled epithelial cells in the visual field. The total number of DAPI-labeled cells in biopsies (n = 5 sections from five biopsies) from HCs (476.7 ± 83.3), IBS-D (383.1 ± 17.47) and IBS-C (447.4 ± 60.23) were comparable (p = 0.31, one-way ANOVA F(2, 12) = 1.293). Labeled tuft cells in human biopsies displayed classic pear-shaped morphology (2) with a large central nucleus and strong Phalloidin-labeled cytoskeletal filaments (**Figure 1A**). The density of tuft cells in IBS-C biopsies (n = 18 sections from six biopsies) was not different to HC biopsies (p >0.05, n = 18 sections from six biopsies, **Figure 1A**). However, the prevalence of tuft cells in IBS-D biopsies (n = 18 sections from six biopsies) was elevated as compared to HC samples (p <0.05, **Figure 1A**).

### Biopsy Secretion of IL-25/hu-17E Is Elevated in IBS-D Samples

As activated tuft cells secrete IL-25 (10), we examined IL-25/hu-17E levels both in local secretions from human biopsies and in the matching plasma samples. IL-25/hu-17E was detected at sub-picomolar concentrations in supernatants from HC biopsies (n = 6) and concentrations were similar in IBS-C patient supernatants (n = 6, p >0.05). However, the concentration of secreted IL-25/hu-17E was elevated in IBS-D supernatants (n = 6, p <0.01, F(2,15) = 7.343, **Figure 1B**). Plasma concentrations of IL-25/hu-17E were also increased in IBS-D samples (p = 0.02, F(2,14) = 5.36, **Figure 1C**).

### Circulating Concentrations of IL-6 and IL-8 Are Altered in IBS Patients

Other inflammatory cytokines, such as IL-6 and IL-8 are reported to be elevated in IBS patients (24, 25). Thus, to confirm the findings from the previous studies, IL-6 was initially compared between plasma from HCs and pooled samples from both IBS-D and IBS-C. We found that circulating IL-6 was elevated in IBS patients (0.976 ± 0.17 pg ml^−1^) as compared to HCs (0.398 ± 0.15 pg ml^−1^, p = 0.06, Student's t-test). When examined by subtype, circulating IL-6 was elevated in IBS-D (1.343 ± 0.35 pg ml^−1^, p = 0.04)) but not IBS-C (0.75 ± 0.06 pg ml^−1^) as compared to HC samples (0.504 ± 0.16 pg ml^−1^, one-way ANOVA F(2,11) = 4.338). Circulating IL-8 concentrations were elevated in pooled IBS plasma samples (12.27 ± 1.06 pg ml^−1^) as compared to HC samples (6.514 ± 0.51 pg ml^−1^, Student's t-test, p = 0.004). However, when examined individually, IL-8 in IBS-C samples (13.44 ± 1.62 pg ml^−1^, p = 0.009) but not in IBS-D samples (11.1 ± 1.3 pg ml^−1^), was elevated by comparison to HC samples (6.5 ± 0.51 pg ml^−1^, one-way ANOVA F(2,14) = 6.799). IL-6 concentrations in secretions from colonic biopsies were not different between HCs (57.87 ± 23.6 pg ml^−1^), IBS-D (40.16 ± 11.45 pg ml^−1^) or IBS-C (28.8 ± 7.7 pg ml^−1^, one-way ANOVA F(2,14) = 0.86, p = 0.45) patients. Secretion of IL-8 from colonic biopsies was also similar in supernatants from HCs (2617 ± 1197 pg ml^−1^) and individuals with IBS-D (926.7 ± 358 pg ml^−1^) and IBS-C (1570 ± 571 pg ml^−1^, one-way ANOVA F(2,15) = 1.31, p = 0.304).

### Colonic Tuft Cell Density Is Elevated in Stress-Sensitive Wistar Kyoto Rats

**Figure 2:**
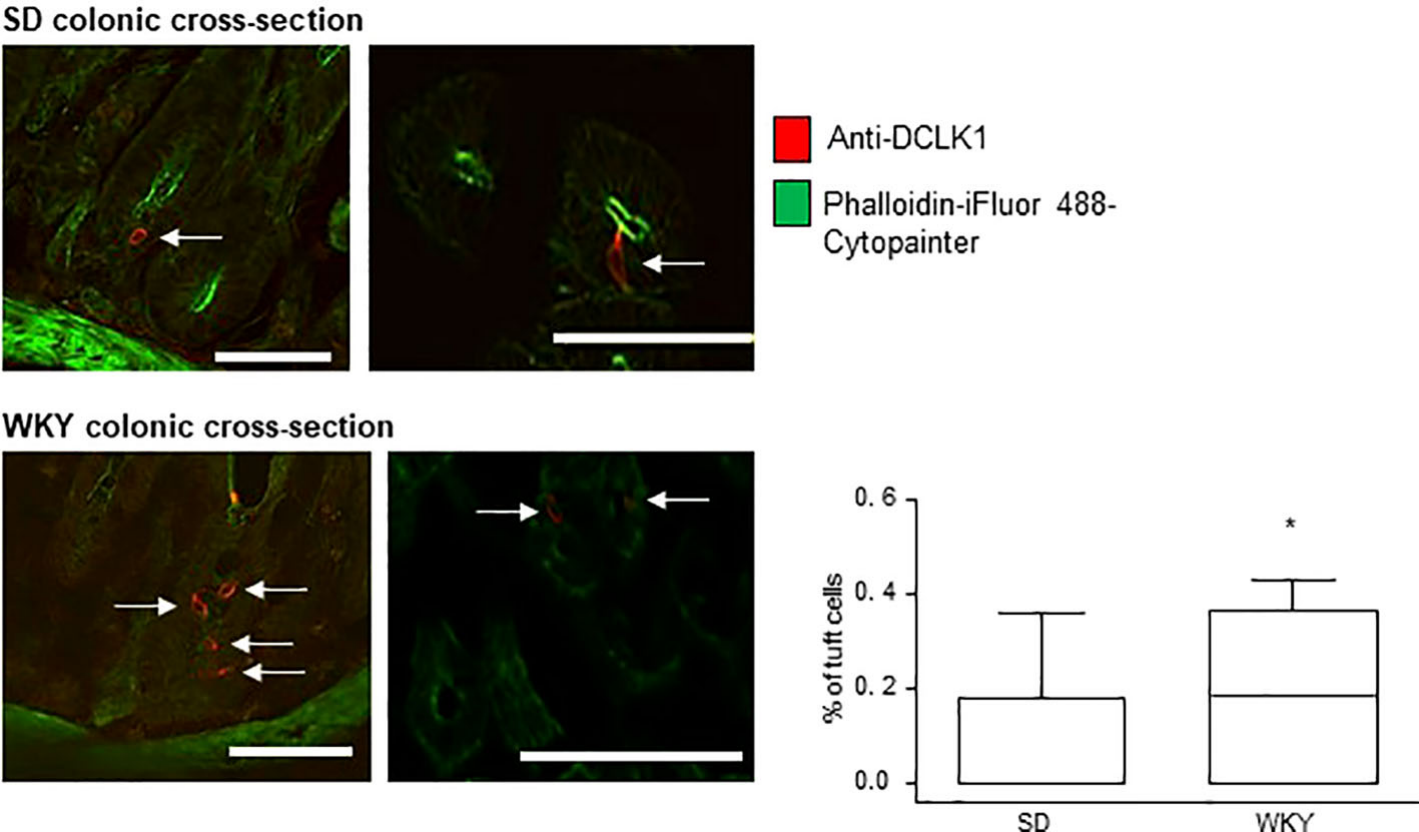
Tuft cell density is increased in Wistar Kyoto (WKY) colons. The representative immunofluorescent images and box and whisker plots of pooled data show the density of DCLK1-labeled tuft cells as a percentage of the total DAPI-stained epithelial cells. Numbers of tuft cells are increased in stress-sensitive WKY rats, which have been validated as an animal model of IBS, as compared to Sprague Dawley control rats. * indicates p <0.05. Scalebar: 50 µm.

Immunofluorescence and confocal microscopy were used to determine the presence and prevalence of tuft cells in the colons of IBS-like Wistar Kyoto (WKY) rats as compared to Sprague Dawley (SD) controls. Triple-labeling with DAPI, anti-DCLK1 (red staining) and a cytoskeletal marker (green labeling) was carried out on colonic cross-sections from SD and WKY rats to determine the density of tuft cells in each rat strain. DCLK1-labeled tuft cells were readily identifiable in cross-sections of both SD and WKY colons (**Figure 2**, red staining, tuft cells indicated by arrows). However, in contrast to tuft cells in human colonic mucosa, rat DCLK1-labeled tuft cells did not strongly express phalloidin-labeled cytoskeletal proteins. They did however, exhibit similar flask shaped morphology (**Figure 2**). The overall number of DAPI-labeled cells was comparable between SD (197.7 ± 49.3, n = 3) and WKY (219.3 ± 22.7, n = 3, p >0.05, Student's t-test) rats. However, the number of tuft cells in WKY rats (n = nine slices from three rats) was increased as compared to SD controls (n = nine slices from three rats; p <0.05, Student's t-test, **Figure 2**).

### Tuft Cell Density Does Not Change in Response to Chronic Stress

**Figure 3:**
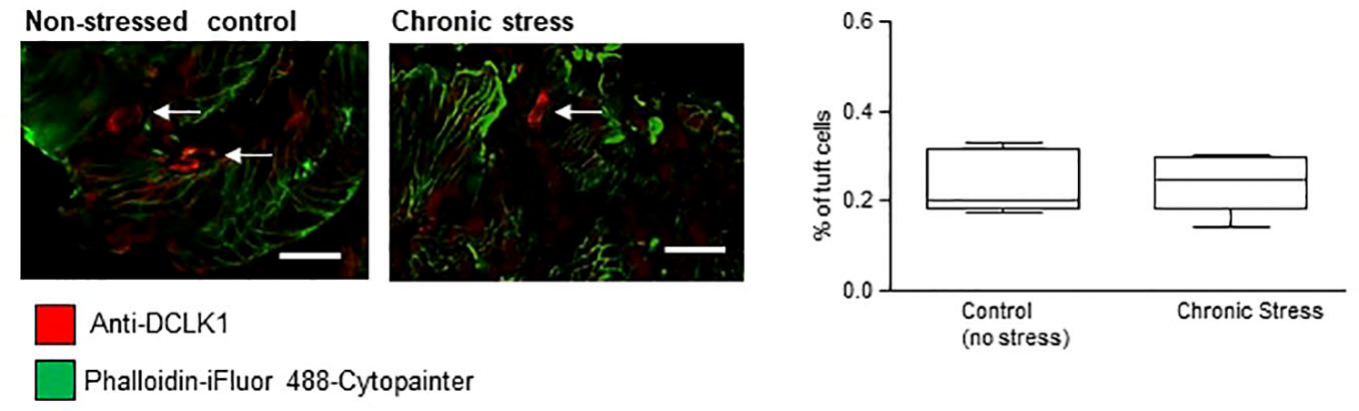
Tuft cell density is not altered by chronic stress. The representative immunofluorescent images and box and whisker plots of pooled data show the density of DCLK1-labeled tuft cells as a percentage of the total DAPI-stained epithelial cells. No difference in the density of tuft cells was detected in colonic samples from mice which had undergone chronic stress. Scalebar: 50 µm.

As sensitivity to stress is a key trait of both WKY rats (26–28) and human IBS (29), we investigated if chronic stress alone impacted on numbers of tuft cells. Colons from male C57/BL6J control mice were compared to mice which had endured 10 consecutive days of chronic social defeat stress (21). The overall number of DAPI-stained epithelial cells in non-stressed control C57/BL6J mice (452.8 ± 109.6, n = 4) was not different to stressed mice (402.5 ± 94.63, n = 4, p >0.05, Student's t-test). DCLK1-labeled (red staining, tuft cells indicated by arrows, **Figure 3**) tuft cells were evident in the colonic mucosa of these mice, but similar to the rat tissue, strong actin labeling was not evident. The density of mucosal tuft cells did not differ between stressed C57/BL6J mice and their non-stressed comparators (n = 15 slices from five mice, p >0.05, Student's t-test, **Figure 3**).

## Discussion

Tuft cells have been proposed as chemosensory sentinels important in the host response to exposure to common eukaryotes, such as helminths and protists (11). Although not well elucidated, mechanisms involving basolateral release of immune or neuromodulatory factors from these cells may result in modulation of gut function (9) through interaction with enteric neural plexi (7, 8). We have examined tuft cell density in colonic mucosal biopsies from patients with IBS, diagnosed in the absence of previous known enteric infection. Increased density of tuft cells was detected only in diarrhea-predominant IBS biopsies. Thus, tuft cell hyperplasia may represent a potential biomarker for this subtype of IBS.

The intestinal profile of IBS patients exhibits lower bacterial diversity that healthy individuals (30, 31). Moreover, transfer of faecal microbiota from IBS-D patients is sufficient to evoke changes in gut function, low-grade inflammation and the expression of anxiety-like behaviors in germ-free mice (32). However, studies focussed only on bacteria cannot explain the heterogeneity of IBS symptomology (33, 34). Given that the human microbiome includes many other non-bacterial microorganisms such as viruses, fungi, archaea and protozoans; other luminal residents have the potential to contribute to the pathophysiology of this functional bowel disorder. Indeed, the mycobiome differs in IBS patients (35) and viral infection has been linked to increased risk of developing IBS (36). The prevalence of protozoans is also increased in IBS patients (37) with some, such as *Dientamoeba fragilis* (38) and *Giardia intestinalis* (39) actually inducing IBS-like symptoms such as abdominal pain and looseness of stools. Chemosensory activation of tuft cells, which are in close proximity to the neuronal plexi that regulate gut function (7, 8), could therefore potentially contribute to IBS symptom manifestation.

Immunofluorescent labeling of tuft cells in mucosal biopsies revealed rare DCLK1-expressing cells which displayed classic pear-shaped morphology (2) and a strong cytoskeletal component. Similar to other studies (1), we found that these tuft cells made up less than 0.4% of DAPI-labeled epithelial cells in control subjects. Biopsies from the distal colon of patients with IBS-C had a similar prevalence of tuft cells to healthy study participants, however, in the absence of any change in total epithelial cell number, IBS-D patients exhibited tuft cell hyperplasia. Although an active helminth infection can induce more than ten-fold increase in tuft cell numbers in the upper intestine (12), our more modest results (< 2 fold) are present in the absence of any documented history of enteric infection.

Helminths and protists evoke a type 2 innate immune response, which is characterized by secretion of ILC2 cytokines. In particular, IL-25, which, in the intestine, is uniquely secreted by tuft cells, is a key signalling molecule secreted in responses to helminth infections (40, 41). IL-25 subsequently stimulates ILC2 to secrete IL-5, IL-9 and IL-13. IL-13 promotes goblet cell hyperplasia and in a feed-forward cycle, tuft cell hyperplasia. Increased goblet cell activity and mucus secretion has been reported in IBS patients (42) and we now provide evidence of tuft cell hyperplasia in IBS-D colonic mucosal samples.

Interestingly, in one study, biopsy-secreted IL-13 was decreased as compared to controls in PI-IBS patients, who had a history of acute gastroenteritis with diarrhea and/or vomiting, (43), although in contrasting results, stimulated lymphocytes from IBS patients secreted more IL-13 as compared to controls, leading the authors to conclude that exposure to bacterial products led to a shift from a Th1 to a Th2 type of cytokine production (44). Our study has detected increased epithelial tuft cell numbers in IBS-D colonic biopsies. Concentrations of local and circulating IL-25 are also elevated in IBS-D samples, which could be related to tuft cell hyperplasia, although no statistical correlation was detected. However, small sample sizes of each IBS subtype could underlie this finding, which is a recognized limitation of the study. Overall, plasma concentrations of IL-25 were notably higher than local secretions, which likely reflects cumulative tuft cell secretion throughout the gut.

We have previously reported changes in cytokine profiles in IBS patients from this geographical region (24, 25), with elevated concentrations of IL-6 and IL-8 in pooled plasma samples from all IBS subtypes. We were able to reproduce these findings in pooled samples, however, subtype-specific analysis determined that IL-6 was only significantly increased in IBS-D subtypes. In contrast, plasma concentrations of IL-8, was elevated only in IBS-C samples. No differences in local concentrations of IL-6 or IL-8 were detected in secretions from colonic biopsies and indeed, there was no statistical correlation between tuft cell density and concentrations of these cytokines. IL-6 and IL-8 both have neurostimulatory actions in the enteric nervous system and also modify gut function (27, 45, 46). While tuft cells have been linked with enteric neuronal function (7, 8), and IL-25 receptor immune-reactivity has been detected in central neurons (47), further studies are needed to explore if this cytokine can modify activity in enteric neurons or gut function. Indeed, if this is found to be the case, it could be through indirect mechanisms, such as through stimulation of mucosal mast cells (48) or other immune cells (10, 11, 49) which are activated by IL-25.

Validated animal models of IBS have been very useful in understanding the pathophysiological changes underlying bowel dysfunction. One such model is the WKY rat, which exhibits visceral hypersensitivity, raised corticosterone in response to a challenge (50) and increased stress-induced defecation (26, 27). Moreover, WKY rats exhibit altered colonic morphology including elevated levels of mucus-secreting goblet cells (26). We determined that distal colonic mucosal sections display DCLK1-immunostained tuft cells with a prevalence of <0.4% in control SD rats. In contrast to the human biopsies, these tuft cells did not express overly strong cytoskeletal proteins. Nonetheless, tuft cell hyperplasia was apparent in the WKY rat model of IBS. In contrast to the human study participants, who may have unknowingly been exposed to parasites resulting in altered bowel function and changes to mucosal cells, the controlled environment in which laboratory animals are maintained, allows us to say with confidence that these animals have not been exposed to helminths or protists. Thus, some other factor may contribute to the increase in tuft cell numbers in WKY rats.

It is generally accepted that psychological stressors are complicit in the onset (51), exacerbation and prolongation of IBS symptoms (52, 53). Stressors can also modify gut morphology and permeability (54). Sensitivity to stress is a key trait in WKY rats, but modified cytokine profiles indicate that immune activation (55), among other factors, also contribute to the overall phenotype. Two groups of C57/BL6J mice, reared under controlled conditions and protected from helminth exposure, were compared to explore if stress alone modifies expression of epithelial tuft cells. A control, non-stressed group was compared to mice which were susceptible to the stress associated with ten consecutive days of chronic social defeat stress. A previously published study using these mice demonstrated that susceptible mice exhibited elevated levels of corticosterone and adrenal gland weight, reflecting dysregulation of the hypothalamic–pituitary–adrenal axis (21). Stressed mice did not exhibit changes in the numbers of colonic epithelial tuft cells, suggesting that activation of the stress axis *per se* does not lead to tuft cell hyperplasia. However, as these mice did display some changes in innate immunity (21), the chronic stressor clearly impacts other systems apart from the stress response.

These studies have determined that tuft cell hyperplasia is evident in patients with IBS-D with no history of enteric infection. A parallel increase in secreted and circulating IL-25 was also observed. Although no statistical correlation was detected between tuft cell density and IL-25 concentrations, this may be detected with a larger sample size. Tuft cell hyperplasia was replicated in a rat model of IBS, which was not exposed to microbes such as helminths or protists. Activation of the stress response, which is central to symptom manifestation and prolongation in functional bowel disturbances, had no impact on tuft cell densities in mice, suggesting that stress, in of itself, does not contribute to tuft cell hyperplasia. The clinical diagnosis of IBS is hampered by the lack of specific biological biomarkers, necessitating a symptom-based diagnosis following exclusion of other organic diseases. Our findings contribute to gathering evidence of subtype-specific changes in intestinal epithelial morphology in IBS patients.

## Data Availability Statement

All data for this study is included in the article/supplementary files or on request from authors.

## Ethics Statement

The studies involving human participants were reviewed and approved by University College Cork Clinical Research Ethics Committee (ECM 4 (r) 010316). The patients/participants provided their written informed consent to participate in this study. The animal study was reviewed and approved by University College Cork animal ethical committee (#2011/015).

## Author Contributions

JA and MC performed the research and analyzed the data. JB contributed human samples. DO'M designed the research study, sourced funding, prepared and reviewed the manuscript.

## Funding

JA was supported by APC Microbiome Ireland, which is funded by Science Foundation Ireland (SFI; grant nos. SFI/12/RC/2273). MC was supported by the Department of Physiology, University College Cork, Ireland.

## Conflict of Interest

The authors declare that the research was conducted in the absence of any commercial or financial relationships that could be construed as a potential conflict of interest.
